# Deletion of arginase 2 attenuates neuroinflammation in an experimental model of optic neuritis

**DOI:** 10.1371/journal.pone.0247901

**Published:** 2021-03-18

**Authors:** Amritha A. Candadai, Fang Liu, Abdelrahman Y. Fouda, Moaddey Alfarhan, Chithra D. Palani, Zhimin Xu, Ruth B. Caldwell, S. Priya Narayanan

**Affiliations:** 1 Clinical and Experimental Therapeutics, College of Pharmacy, University of Georgia, Augusta, GA, United States of America; 2 Culver Vision Discovery Institute, Augusta University, Augusta, GA, United States of America; 3 Charlie Norwood VA Medical Center, Augusta, GA, United States of America; 4 Vascular Biology Center, Augusta University, Augusta, GA, United States of America; 5 Department of Cellular Biology and Anatomy, Augusta University, Augusta, GA, United States of America; Instituto Cajal-CSIC, SPAIN

## Abstract

Vision impairment due to optic neuritis (ON) is one of the major clinical presentations in Multiple Sclerosis (MS) and is characterized by inflammation and degeneration of the optic nerve and retina. Currently available treatments are only partially effective and have a limited impact on the neuroinflammatory pathology of the disease. A recent study from our laboratory highlighted the beneficial effect of arginase 2 (A2) deletion in suppressing retinal neurodegeneration and inflammation in an experimental model of MS. Utilizing the same model, the present study investigated the impact of A2 deficiency on MS-induced optic neuritis. Experimental autoimmune encephalomyelitis (EAE) was induced in wild-type (WT) and A2 knockout (A2^-/-^) mice. EAE-induced cellular infiltration, as well as activation of microglia and macrophages, were reduced in A2^-/-^ optic nerves. Axonal degeneration and demyelination seen in EAE optic nerves were observed to be reduced with A2 deletion. Further, the lack of A2 significantly ameliorated astrogliosis induced by EAE. In conclusion, our findings demonstrate a critical involvement of arginase 2 in mediating neuroinflammation in optic neuritis and suggest the potential of A2 blockade as a targeted therapy for MS-induced optic neuritis.

## Introduction

Multiple Sclerosis (MS) is a chronic, autoimmune, inflammatory, and neurodegenerative disease of the central nervous system (CNS) [1, 2]. This disorder affects approximately 400,000 people in the United States and 2.1 million people worldwide [3], with a higher incidence rate in women [4, 5]. Visual dysfunction due to optic neuritis is a common complication faced by approximately 20% of MS patients [6, 7]. Optic neuritis caused by inflammation of the optic nerve is characterized by thinning of the nerve fiber layer, retinal ganglion cell (RGC) loss, and axonal degeneration [8–11]. Symptomatically, a patient experiences unilateral optic neuritis that may present with acute pain in the retroorbital and/or periorbital regions, blurred vision, color vision deficits, and ultimate vision loss [12]. Current medications available for MS-induced optic neuritis such as i.v. methylprednisolone or other oral steroids are only partially effective [13]. They provide symptomatic benefits but have limited impact on the neuroinflammatory pathology of the disease. An agent with anti-inflammatory and neuroprotective effects may offer advantages over existing therapies in reducing MS disability.

Experimental autoimmune encephalomyelitis (EAE) is an established experimental murine model for MS studies [14]. Previous studies have demonstrated that EAE mice develop retinal damage, RGC loss, and optic neuritis mediated by an inflammatory cascade and neurodegeneration [15–18]. Oxidative stress is a key mechanism implicated in MS and EAE progression [19, 20]. This pathological mechanism may be driven by several pathways such as activation of microglia/macrophages and altered nitric oxide synthase (NOS) that contribute to the generation of reactive species like reactive oxygen species (ROS), reactive nitrogen species (RNS). Enhanced expression of inducible NOS (iNOS) and activated microglia were found in MS lesions and EAE animals [21–23]. Increased arginase activity also correlated with iNOS expression in the brain and spinal cord of EAE animals [24].

A recently published study from our laboratory demonstrated a retinal protective effect of Arginase 2 (A2) deletion in the EAE model [25]. A significant increase in neuronal survival was accompanied by a reduction in the expression of proinflammatory molecules and decreased glial activation in the retina. EAE-induced motor deficits were also decreased in response to A2 deletion. The goal of our current study is to further characterize the protective effects of A2 deletion in EAE-induced optic nerve degeneration. Utilizing a combination of immunofluorescence staining and imaging techniques, this study investigated the impact of A2 deletion on EAE-induced inflammatory changes and axonal pathology in the optic nerve.

## Materials and methods

### Animals and induction of EAE

Wild-type (WT) and arginase 2 knockout (A2^−/−^) mice on a C57BL/6J background were maintained in our animal facility and used for this study. This study was conducted in strict accordance with the ‘ARVO Statement for the Use of Animals in Ophthalmic and Vision Research’. All procedures were performed according to the approved institutional guidelines (Animal Welfare Assurance no. A3307–01) and adhered to the Public Health Service Policy on Humane Care and Use of Laboratory Animals (revised July 2017). The protocol was approved by the Institutional Animal Care and Use Committee the Augusta University (Protocol Number: 2016–0823). All efforts were made to assure the minimum possible suffering during experimental procedures. Mice were euthanized by overdose with a ketamine/xylazine cocktail. The EAE induction kit (Hooke Laboratories, Lawrence, MA, cat. no. EK-2110) was utilized for chronic EAE induction [25]. On day 0, mice in the EAE group received subcutaneous injections of myelin oligodendrocyte glycoprotein (MOG_35–55_) peptide emulsion (200 μL, 200 μg/mouse) along with Complete Freund’s Adjuvant (CFA, killed *M*. *tuberculosis* H37Ra, 400 μg/μL). Injections of pertussis toxin (PTX, 100 ng in 50 μL PBS) were administered intraperitoneally to each mouse 2h post-immunization on day 0 and day 1. The control group received the same treatment excluding the peptide injection. Four groups were included in the study; a) **WT control**: WT mice immunized with CFA alone and PTX, b) **WT EAE**: WT mice immunized with MOG in CFA and PTX, c) **A2**^**−/−**^ **control**: A2^−/−^ mice immunized with CFA and PTX, and d) **A2**^**−/−**^ **EAE**: A2^−/−^ mice immunized with MOG in CFA and PTX. Neurological deficits were assessed daily using a blinded scoring method according to the conventional grading system as described in our previous study [25]. Briefly, 0, no disease; 1, complete loss of tail tonicity; 2, partial hind limb paralysis (uneven gate of hind limb); 3, complete hind limb paralysis; 4, complete hind and forelimb paralysis; and 5, moribund or dead. Animals displaying paralysis on four limbs and/or weight loss more than 15% were sacrificed. Soft food was provided in the cage for paralyzed mice.

### Optic nerve isolation and sectioning

Following 60 days post-induction, mice were euthanized by overdose with ketamine/xylazine cocktail, and optic nerves were removed, post-fixed in 4% paraformaldehyde, cryoprotected with 30% sucrose, embedded in optimum cutting temperature (OCT) compound and stored at − 80°C. Longitudinal sections of the optic nerve (10 μm) were prepared using a cryostat and mounted on gelatin-coated glass slides. Sections were preserved at − 80°C for immunostaining.

### Hematoxylin and Eosin (H&E) staining and analysis of cellular infiltration

Optic nerve sections were subjected to H&E staining at Augusta University histology core. Images were taken using Zeiss Axioplan 2 microscope and the analysis was performed using the “point tool” function of NIH ImageJ software (National Institutes of Health, USA).

### Immunofluorescence staining of optic nerve

Optic nerve cryo-sections were acclimatized to room temperature and rehydrated using wash buffer 1X phosphate buffered saline (PBS). Excess buffer was drained, and tissue sections were surrounded with a hydrophobic barrier using ImmEdge® Pen (Vector Laboratories). Permeabilization was achieved using 1% Triton X-100 in PBS for 10 mins, followed by a PBS wash cycle and blocking (10% donkey serum for 1h) at room temperature. Sections were washed with PBS and incubated with respective primary antibodies (Table 1) overnight. The next morning, sections were washed, followed by incubation with the appropriate secondary antibodies (Table 1) for 2h. Sections were washed (1X PBS), dried, and covered with mounting medium for imaging.

**Table 1:** Details of primary and secondary antibodies used.

Antibody used	Catalogue No.	Company	Dilution
Arginase 1	610709	BD Biosciences, San Jose, CA, USA	1:200
CD16/32	553142	BD Biosciences, San Jose, CA, USA	1:100
CD 86	ab213044	Abcam, Cambridge, MA, USA	1: 500
F4/80	ab6640	Abcam, Cambridge, MA, USA	1:200
GFAP	Z033429-2	Dako, Carpinteria, CA, USA	1:200
Iba1	019–19741	Wako, Richmond, VA, USA	1:200
MBP	MAB386	MilliporeSigma, St. Louis, MO, USA	1:100
SMI 32	801701	BioLegend, San Diego, CA, USA	1:200
Secondary antibody used			
Donkey anti-Rabbit IgG (H+L) Polyclonal Secondary Antibody, Alexa Fluor 488	A21206	Invitrogen™, Waltham, MA, USA	1:500
Donkey anti-Rat IgG (H+L) secondary Antibody, Alexa Fluor 488	A21208	Invitrogen™, Waltham, MA, USA	1:500
Donkey anti-Mouse IgG (H+L) Polyclonal, Secondary Antibody, Alexa Fluor 555	A31570	Invitrogen™, Waltham, MA, USA	1:500

### Imaging and quantification

Images were captured using Keyence fluorescence microscope (BZ-X800, Itasca, Illinois, USA) and/or confocal microscope (LSM 780; Carl Zeiss, Thornwood, NY, USA). Staining appeared homogenous throughout the optic nerve unless stated otherwise in the results. For quantification, a minimum of two sections per optic nerve were utilized for each antibody. A minimum of three non-overlapping fields per section were imaged using confocal microscope for quantification, resulting in a minimum of 6 images per mouse per antibody. A minimum of 5 animals per group were included for each study.

Quantification Iba1 positive cells based on morphology was performed using ImageJ software. Briefly, 40X confocal images (maximum intensity projection) were converted to 8-bit grey scale and a threshold was applied to track the cells. The cells with enlarged soma size were selected and quantified using the “Analyze particle” function (pixel size: 500-infinity and circularity: 0.10–1.00). Quantification of F4/80 (EGF-like module containing mucin-like hormone receptor-like 1), CD 86, A1 and CD 16/32 was performed on 40X images using the “point tool” function of ImageJ software. Fluorescence intensity was measured as integrated density using ImageJ software, and the mean values were calculated and expressed as the fluorescence intensity per field of view for each marker used. The area of fields measured per marker (SMI 32 (Neurofilament H (non-phosphorylated), MBP (Myelin Basic Protein) and GFAP (Glial Fibrillary Acidic Protein)) was maintained uniformly throughout all groups and values were normalized relative to the percentage of WT control group.

### Statistical analysis

All statistical analyses were performed with GraphPad Prism 7 (GraphPad Software Inc., La Jolla, CA). Outliers were eliminated using Grubbs’ outliers test at a significance level of 0.05. Two-way ANOVA followed by Tukey’s multiple comparisons test was employed to analyze the groups. A p<0.05 was defined as statistically significant. Results are presented as Mean ± SEM.

## Results

### A2 deletion reduced EAE-induced cellular infiltration in the optic nerve

**Fig 1:**
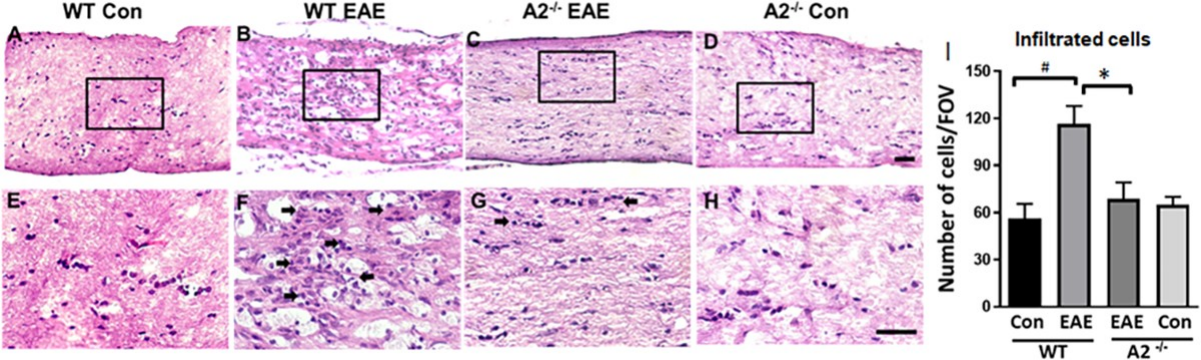
Changes in EAE-induced cellular infiltration in the optic nerve. A-D) Representative images of H&E stained sections from control and EAE optic nerves. Increased infiltration was evident in WT EAE sections, while A2 deletion markedly reduced this effect. E-H) High magnification images of boxed regions demonstrate EAE-induced cellular infiltration. Arrows indicate clusters of infiltrated cells. I) Quantification of infiltrated cells using ImageJ. Data are presented as Mean ± SEM. #p<0.01 WT EAE vs WT Con and *p<0.05 A2^-/-^ EAE vs WT EAE. Number of animals used: 8(WT Control);12 (WT EAE); 9 (A2^-/-^ EAE); 7 (A2^-/-^ Control). Representative images are presented. Scale bar 50 μm.

**Fig 2:**
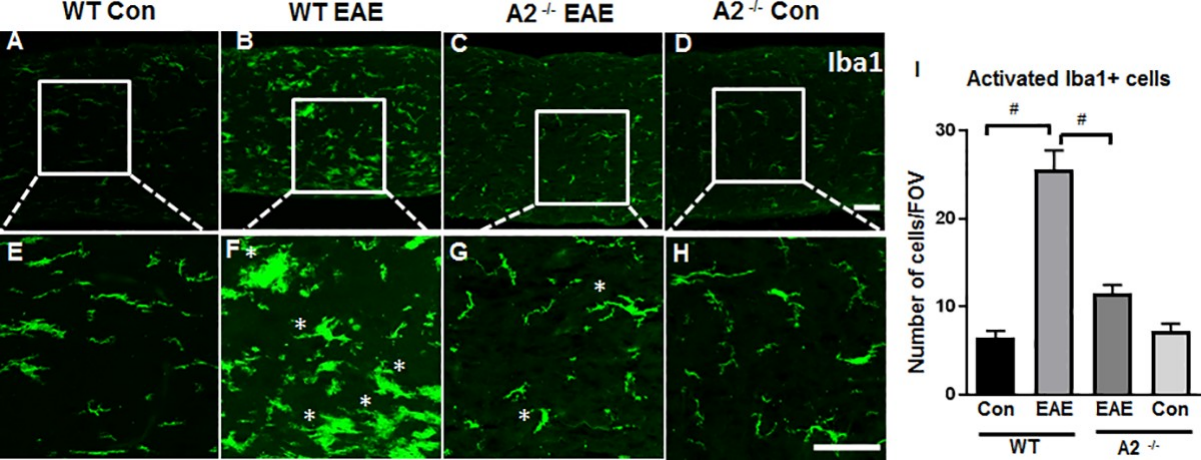
EAE-induced microglial/macrophage activation is ameliorated in the optic nerve of A2 knockout mice. A-D) Immunofluorescence images of Iba1 labeled optic nerve sections showing increases in cells with activated morphology in response to EAE induction. The deletion of A2 significantly reduced this effect. E-H) Magnified images of the boxed regions demonstrate changes in the morphology of Iba1 positive cells and the effects of A2 deletion. Asterisks indicate Iba1 positive cells with activated morphology following EAE induction. I) Quantification of Iba1 positive cells with activated morphology. Data are presented as Mean ± SEM. #p<0.01 WT EAE vs WT Con and # p<0.01 A2^-/-^ EAE vs WT EAE. Number of animals used: 8(WT Control);12 (WT EAE); 9 (A2^-/-^ EAE); 7 (A2^-/-^ Control). Representative images are presented. Scale bar 50 μm.

Histological analysis of optic nerve sections demonstrated increased cellular infiltration in the EAE optic nerves (Fig 1). WT EAE optic nerve sections showed hypercellularity compared to WT controls (Fig 1A and 1B). However, EAE-induced cellular infiltration was markedly reduced in the A2^-/-^ optic nerve samples (Fig 2C). Magnified images (Fig 1E–1H) show increased clusters of infiltrated cells in the WT EAE optic nerve compared to the A2^-/-^ EAE and the control samples. Quantification of the infiltrated cells presented in Fig 1I demonstrated a two-fold increase in the WT EAE optic nerve compared to the WT control. EAE optic nerves from A2^-/-^ mice showed significantly reduced levels of cellular infiltration (Fig 1I).

### EAE-induced microglial/macrophage activation is reduced by A2 deletion

**Fig 3:**
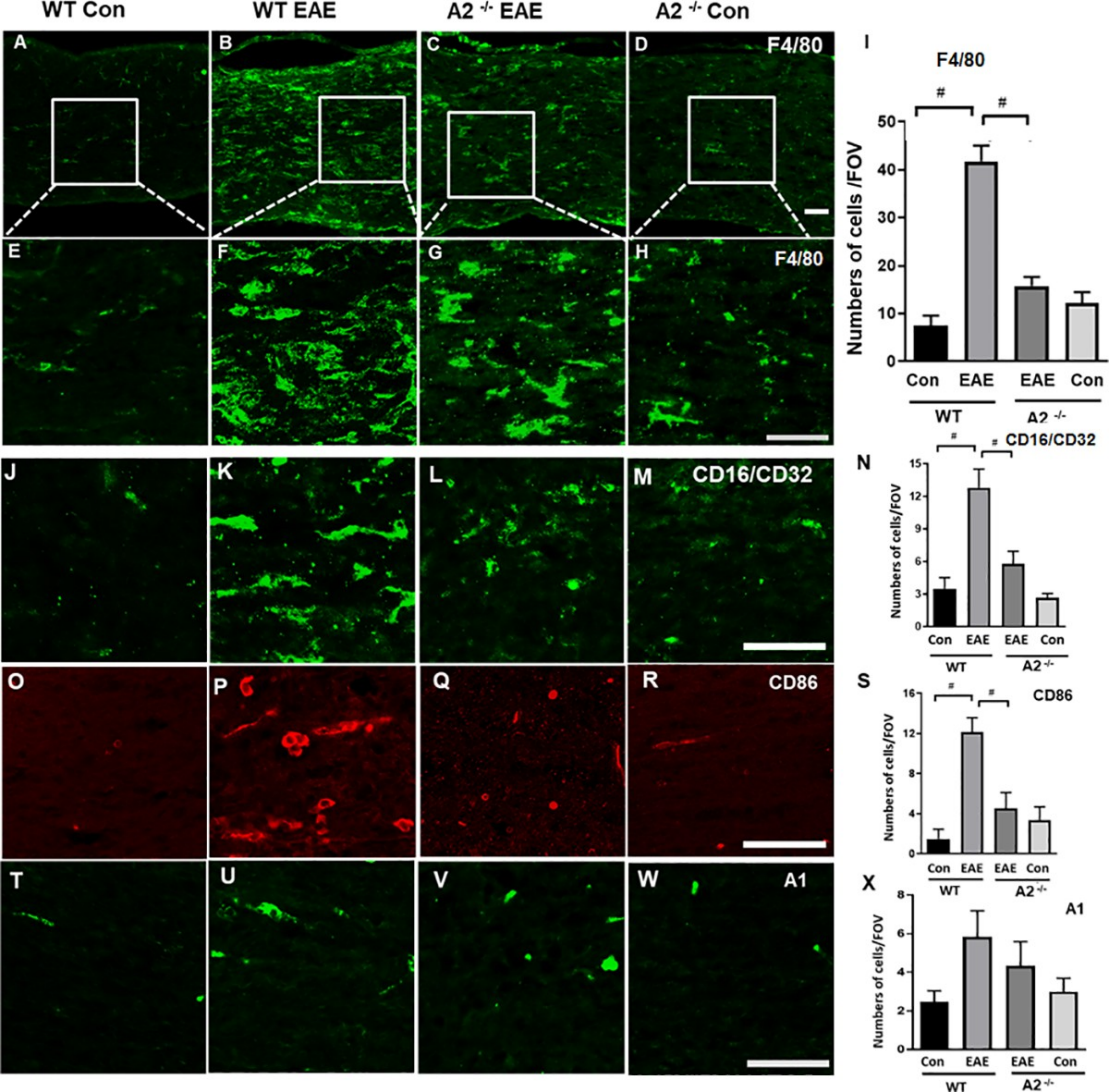
A2 deletion suppressed EAE-induced upregulation of F4/80 cells. A-D) Representative confocal images of optic nerve sections immunostained using F4/80 antibody. WT EAE optic nerves showed an increase in macrophage/monocyte, while A2 deletion greatly suppressed this effect. E-H) Magnified images of boxed areas represent increase in the presence of F4/80 positive cells induced by EAE. I) Quantification of F4/80 positive cells demonstrate the impact of A2 deletion on the number of macrophages induced by EAE. Optic nerve sections stained using antibodies against CD 16/32 (J-M), and CD 86 (O-R) and quantification (N&Q) showed upregulation of M1 macrophages in WT EAE samples which was significantly reduced by the A2 deletion. Studies using an M2 marker, A1, did not show any significant changes across the groups (T-W) Number of animals used in the study: Number of animals used: 8(WT Control);12 (WT EAE); 9 (A2^-/-^ EAE); 7 (A2^-/-^ Control). Representative images are presented. Scale bar 50 μm. Data are presented as Mean ± SEM. #p<0.01 WT EAE vs WT Con and # p<0.01 A2^-/-^ EAE vs WT EAE.

Immunofluorescence staining using Iba1 (Ionized calcium-binding adaptor molecule 1) and F4/80 (EGF-like module-containing mucin-like hormone receptor-like 1) antibodies were utilized to demonstrate activation of microglia and macrophages in response to EAE (Figs 2 and 3). Morphologically, resting microglia appear elongated with well-defined processes and activated microglia show a more compact ameboid appearance [26]. As shown in Fig 2A, 2B, 2E and 2F, WT EAE optic nerves demonstrated increased Iba1 positive cells with activated morphology compared to the WT control group. Deletion of A2 ameliorated these EAE-induced changes in the optic nerve (Fig 2C and 2G). Quantification of the Iba1 positive cells with activated morphology (i.e. enlarged cell body) (Fig 2I) shows a significant increase in the WT EAE group as compared with the WT Control (^#^p<0.01), and this change was significantly prevented in the A2^−/−^EAE group (*p<0.01). A2^-/-^ control optic nerves did not show any noticeable alterations in the morphology of Iba1 positive cell as compared with the WT control nerves.

F4/80 (EGF-like module-containing mucin-like hormone receptor-like 1) is a marker for macrophagic/microglia cells [27, 28]. In our study, immunostaining of the optic nerve sections was used to demonstrate EAE-induced changes using F4/80 positive macrophage/microglia cells. WT EAE optic nerves demonstrated an increased level of F4/80 cells in comparison to WT Con (Fig 3A, 3B, 3E and 3F). EAE-induced increase in F4/80 positive cells was downregulated in response to A2 deletion (Fig 3C and 3G). These observations are supported by the quantification studies indicating a significant increase in F4/80 positive cells (Fig 3I) in WT EAE versus WT Con group (^#^p<0.01), which was significantly suppressed in the A2^−/−^EAE group (*p<0.01).

Macrophages are known to exist in two major types, the pro-inflammatory M1 and anti-inflammatory M2 [29] and can transition between the phenotypes depending on the microenvironment [30]. Further characterization using markers for M1 and/or M2 phenotypes demonstrated upregulation in the M1-like macrophage phenotype (studied by CD 16/CD 32 and CD 86 markers) in WT EAE optic nerve compared to WT Control (Fig 3J and 3K, versus O and P, respectively). This alteration was prevented in the A2^-/-^ EAE samples (Fig 3L and 3Q). Quantification studies show that the number of cells positive for CD 16/CD 32 (Fig 3N) and CD 86 (Fig 3S) was significantly increased in WT EAE optic nerves compared to the WT control group (^#^p<0.01), while these changes were prevented in the A2^−/−^ EAE group (*p<0.05). Immunostaining experiments using anti-Arginase 1, an M2 marker, did not show any difference between the two EAE groups (Fig 3T–3X).

### Evaluation of EAE-induced axonal degeneration and demyelination

**Fig 4:**
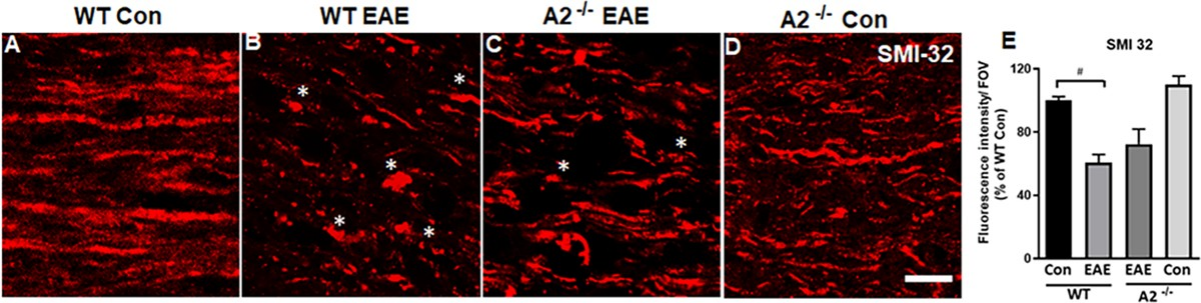
Deletion of A2 reduced EAE-induced axonal degeneration. A-D) Confocal images of optic nerve sections from WT EAE mice demonstrate disorganized and damaged axons in the EAE optic nerves. Asterisks (*) indicate areas of axonal damage. E) Quantification of SMI 32 fluorescence intensity using ImageJ. Number of animals used: 8(WT Control);12 (WT EAE); 9 (A2^-/-^ EAE); 7 (A2^-/-^ Control). Representative images are presented. Data are presented as mean ± SEM. #p<0.01 WT EAE vs WT Con and A2^-/-^ EAE vs WT EAE is non-significant. Scale bar 20 μm.

Axonal degeneration is a characteristic feature of EAE. Immunnolabeling for SMI 32 (marker of neurofilament proteins) was employed to assess the axonal changes in response to EAE. The SMI 32 antibody has shown to label RGC cell bodies and axons [31–33]. Morphological changes suggesting degeneration/disorganization of axons were evident in the EAE optic nerves. Immunolabeling of SMI32 in optic nerves from the control group demonstrated long fibers arranged in parallel, while degenerative changes and structural distortions such as shorter and transected axons and presence of spheroids were observed EAE optic nerves (Fig 4A–4D). These alterations were reduced in the optic nerves lacking A2. Quantification data presented in Fig 4E demonstrate the significantly reduced levels of SMI32-positive neurofilaments in WT EAE optic nerve compared to WT control (^#^p<0.01), however, the improvement observed in A2^-/-^ EAE was not statistically significant with WT EAE group.

**Fig 5:**
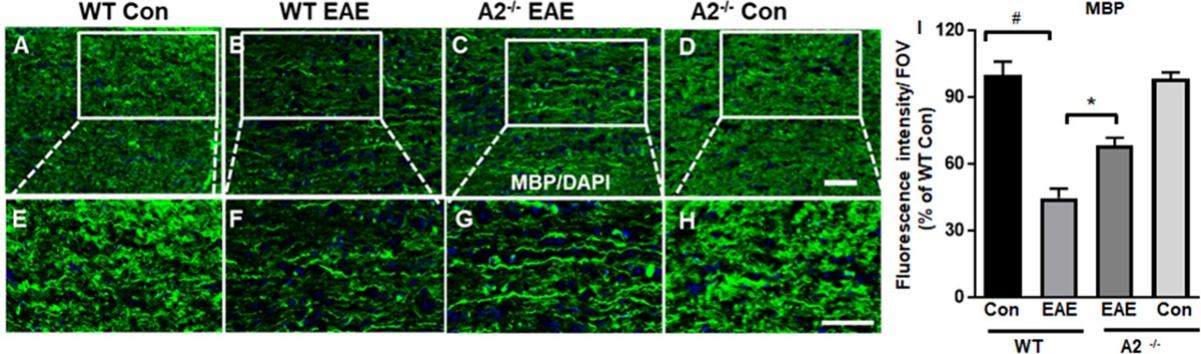
A2 deletion protected against EAE-induced demyelination. A-D) Representative images showing MBP staining. E-H) Higher magnification images from boxed areas showing the loss of myelin in WT EAE optic nerves while A2 deletion reduced the EAE-induced-demyelination. I) Quantification of immunofluorescence intensity of MBP. Number of animals used: 8(WT Control);12 (WT EAE); 9 (A2^-/-^ EAE); 7 (A2^-/-^ Control). Representative images are presented. Data are presented as mean ± SEM. #p<0.01 WT EAE vs WT Con and *p<0.05 A2^-/-^ EAE vs WT EAE. Scale bar 50 μm.

Along with the degeneration of axons, demyelination is another major characteristic seen in MS and EAE. Immunofluorescence using myelin basic protein (MBP) was used to analyze the extent of demyelination in the EAE optic nerves (Fig 5). WT EAE optic nerves showed greater myelin loss (studied by reduced MBP levels) when compared with WT control nerves (Fig 5A, 5B, 5E and 5F). This loss was suppressed in A2 deficient EAE optic nerves (Fig 5C and 5G) suggesting reduced demyelination in response to EAE. Quantification data presented in Fig 5I demonstrate the significantly reduced MBP (fluorescence intensity) levels in WT EAE optic nerve compared to WT con (^#^p<0.01), while this decrease was significantly abrogated in A2^-/-^ EAE optic nerves (*p<0.05).

### EAE-induced astrogliosis was reduced in A2-/- optic nerve

**Fig 6:**
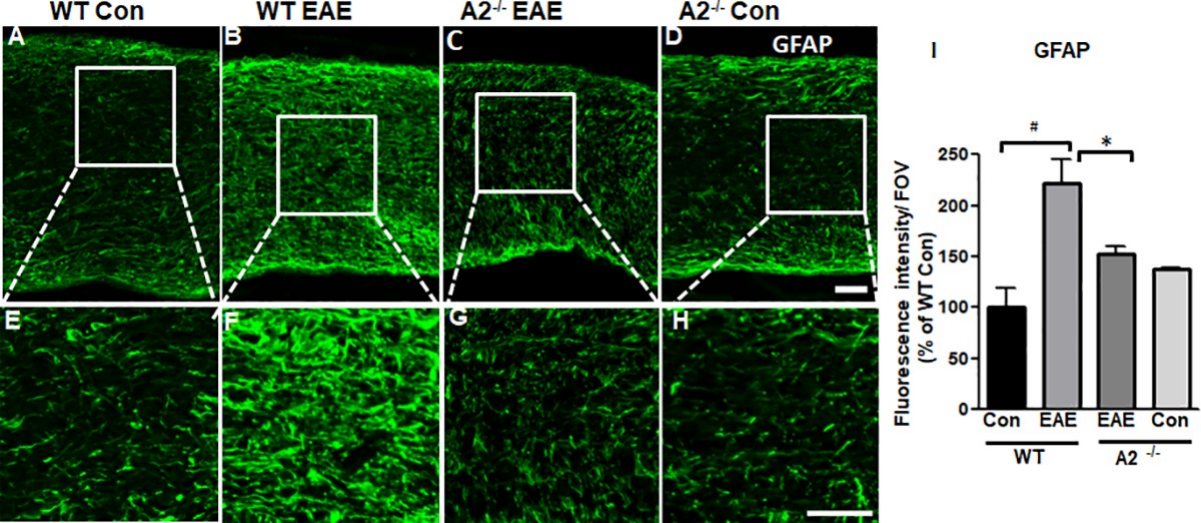
EAE-induced astrogliosis is reduced in A2 deficient optic nerves. **A-D)** Representative confocal images optic nerve sections demonstrate the activation of astrocytes (labeled by GFAP antibody) in the WT EAE optic nerves while the deletion of A2 significantly suppressed the extent of astrogliosis. E-H) Magnified images of respective boxed areas in A-D demonstrate hypertrophic astrocytes. I) Quantification of glial activation in optic nerve sections of different groups. Scale bar 50 μm. Number of animals used: 8(WT Control); 12 (WT EAE); 9 (A2^-/-^ EAE); 7 (A2^-/-^ Control). Representative images are presented. Data are presented as mean ± SEM. #p<0.01 WT EAE vs WT Con and *p<0.05 A2^-/-^ EAE vs WT EAE.

Changes in the extent of astrogliosis were evaluated using GFAP (Glial Fibrillary Acidic Protein) antibody. EAE induction resulted in increased levels of reactive astrocytes as shown by increased immunoreactivity for GFAP in the WT EAE optic nerves compared to WT Con optic nerves (Fig 6A, 6B, 6E and 6F). This was largely prevented by A2 deletion (Fig 6C and 6G). Quantification of GFAP intensity confirmed the significant increase in WT EAE as compared with WT Con (#p<0.01). This increase was suppressed with A2 deletion in A2^−/−^ EAE in comparison to WT EAE (*p<0.05) (Fig 6I).

## Discussion

MS pathogenesis is dominated by the combined action of inflammation and demyelination leading to axonal degeneration. In the present study, we investigated the impact of Arginase 2 (A2) deletion in the degeneration of the EAE optic nerve. To the best of our knowledge, this is the first report determining the impact of A2 deletion in regulating EAE-induced inflammation, axonal damage, and myelin loss in the optic nerve. As described in our previous study [25], EAE-induced motor deficits were milder in A2^-/-^ EAE mice as demonstrated by the lower clinical scores throughout the induction period. The initial signs of paralysis indicated by the loss of tail tonicity started at day 9 in WT EAE mice, and the clinical signs of EAE increased gradually. In A2^-/-^ mice induced with EAE, appearance of the initial signs of paralysis was delayed (beginning at day 11) and the clinical scores were significantly lower compared to WT EAE mice [25].

MS-induced optic neuritis is characterized by inflammation of the optic nerve along with axonal degeneration and RGC loss [8–11]. Infiltration of inflammatory cells into the EAE optic nerve has been reported by several studies [15, 34–36]. These mainly include infiltration of T cells (CD^4+^), and macrophages into the CNS during EAE progression [15, 35, 37–39]. Previous studies have shown increased arginase activity, NO production, and methylated arginine derivatives in the EAE brain and spinal cord tissues suggesting their strong involvement in the inflammatory and neurodegenerative pathways of disease progression [24, 40, 41]. Both A1 and A2 isoforms of arginase are also known to be expressed in the brain and retina [42]. Previous studies from our group have demonstrated that A2 deletion reduces retinal inflammation in EAE and other models of retinal injury [43–45]. Disruption of the blood-brain barrier (BBB) and subsequent cellular infiltration into the CNS is well-established in MS pathology. This process is characterized by the surge of T lymphocytes and monocyte-derived macrophages that cause demyelinating CNS lesions [46, 47]. Microglia and macrophages are known to interact with T-cells and modulate their function in EAE [48]. Likewise, microglial activation and macrophage/T-cell influx have been validated in the optic nerve in previous EAE studies [49, 50]. Our studies show a reduction in cellular infiltration and cluster formation in A2^-/-^ EAE optic nerves.

Previous studies in the EAE model have shown microglial as well as macrophagic autoimmune response and subsequent activation of the inflammatory cascade in the spinal cord, brain, and optic nerve [16, 51]. Consistent with our previous observations in the retina of EAE mice [25], our current study showed that EAE-induced inflammatory responses were significantly reduced in the optic nerves of EAE mice lacking the A2 gene. This was further confirmed by our findings that demonstrated reduced Iba1^+^ and F4/80^+^ cells in A2^-/-^ EAE optic nerves. Our results further showed that the presence of M1-like pro-inflammatory macrophages are markedly reduced in response to A2 deletion. The definite roles of macrophage populations remain controversial and are yet to be elucidated. Cells of the monocyte/macrophage lineage can shift between activation stages in response to cues in the local microenvironment [52, 53]. Arginase 1 (A1) is a marker for the M2-like pro-reparative macrophage phenotype [54–56], and our previous study has shown an increased A1 mRNA level in the A2^-/-^ EAE retina compared to the WT EAE retina [25]. However, no significant changes were observed in A1 positive cells in the EAE optic nerves at 60 days. Previous studies showed A1 to be strongly upregulated in the spinal cord of EAE models [57, 58]. Others have demonstrated the upregulation of A1 positive myeloid cells during EAE progression with inverse correlation with disease severity [59–61]. In the present study, we investigated A1 positive cells at a later time point (60 days post induction) and that could be the reason for not observing any differences.

Several studies have demonstrated axonal damage and demyelination in EAE-induced optic neuritis [15, 36, 62, 63]. Activated microglia and infiltrated macrophages are targeted towards demyelination and axonal injury. Disrupted axonal deposits and swelling of mitochondria and other organelles is a distinctive morphological feature of injured axons [63–65]. In the present study, axonal damage was studied using immunolabeling with an SMI 32 antibody (which reacts with a nonphosphorylated epitope in neurofilament H of most mammalian species). SMI 32 labels neuronal cell bodies, dendrites, and some thick axons in the central and peripheral nervous systems (manufacturer’s datasheet). In the retina, this antibody has been shown to immunolabel the cytoskeleton of medium and large ganglion cells, as well as ganglion cell axons of mouse, rat and cat retina [66–68]. SMI 32 has been used a marker to study abnormalities in the brain and spinal cord in experimental models of MS [69–72], as non-phosphorylated neurofilaments are a predominant feature in damaged axons. However, in our study the fluorescent intensity of SMI 32 immunostaining was reduced in the EAE optic nerves. This could be due to differences in the antibody we used or the time point of our analysis as compared with the previous studies. A recent study (using the same antibody resource) reported an increase in SMI 32 in EAE optic nerves at an early stage of disease (16 days post induction (DPI)), while the expression was significantly downregulated at a later stage (42 DPI) of EAE progression [17]. These results are consistent with our observation where SMI 32 was decreased in EAE optic nerves at 60 DPI. Some authors have suggested that the changes in SMI 32 levels result from impaired axonal transport at the peak of EAE followed by axonal loss at later stages of the disease [17]. An earlier study also reported increased SMI 32 levels in the EAE optic nerves at 14 days after the induction [73]. Our future studies will address the time dependent changes in SMI 32 expression and axonal degeneration in the WT and A2 deficient EAE optic nerves. The protective effects demonstrated by A2^-/-^ optic nerves could be due to cumulative anti-inflammatory and neuroprotective effects mediated by A2 deletion. A previous study has demonstrated that A1 can promote axonal regeneration via increased spermidine synthesis [74]. A marked reduction of EAE-induced myelin loss in spinal cord lesions by A2 deletion was revealed by Choudry et al. [75]. In our study, we observed that damage to the axonal filaments and myelin fibers in EAE optic nerves was limited in the A2^-/-^ mice. Our future studies will investigate the integrity of myelin sheath using electron microscopy.

Astroglial hypertrophy is an important indication of tissue injury and could be resulting from disruption of the astrocyte-oligodendrocyte network. Physiologically, astrocytes maintain homeostasis of a healthy CNS by upholding a constant anti-inflammatory and protective environment [76, 77]. However, injury-induced reactive astrocytes are a source of increased reactive oxygen species (ROS) formation and pro-inflammatory cytokines [78]. Previous reports from our group have demonstrated that lack of A2 reduced activation of Müller glial cells in retinal injury models [43, 44]. Our study reveals significantly reduced gliosis in the A2^-/-^ EAE optic nerve, which further confirms the protective effects mediated by A2 deletion in the CNS and its potential as a therapeutic target. However, one limitation is the lack of a specific inhibitor for arginase 2. Hence, proper design of more selective and cell-targeted inhibitors is needed to validate A2 as a therapy for the disease.

Overall, our present study shows the impact of A2 deletion in preserving neuroimmune functions in the retina and optic nerve. Studies from other laboratories have demonstrated the involvement of arginase signaling in MOG-induced EAE mouse models [56, 59, 75, 79]. Involvement of A1 in EAE models is previously reported. Levels of A1 mRNA are elevated in spinal cord samples from EAE mice [61]. A1 positive myeloid cells have been found to be upregulated during the progression of EAE [59–61] and their presence was shown to be inversely correlated with the severity of disease with greater expression at the earlier stage [61, 80]. These A1 expressing cells, especially the myeloid-derived suppressor cells are hypothesized to inhibit T-cell activation and therefore, may dampen the autoimmune-mediated demyelination in EAE [81, 82]. A report by Xu et al provided data showing a less severe EAE phenotype in mice treated with the arginase inhibitor, amino-6-boronohexanoic acid (ABH) [57]. However, ABH inhibits both arginase isoforms and may be protective due to inhibition of A2. A possible interaction between the two arginase isoforms in EAE is unknown and yet to be elucidated. Oxidative stress mediated by the generation of ROS/RNS and activation of inflammatory cascade evidently contributes towards MS as well as EAE pathogenesis [21–23]. Increased activity of iNOS and arginase levels in EAE animals also demonstrates their involvement [24]. Correspondingly, our group has previously shown that A2 deletion is protective against neuronal apoptosis by suppressing polyamine oxidation via the ornithine/polyamine pathway [44]. These findings portray the significance of arginase and associated signaling molecules (NOS, polyamines, ROS, RNS) in the neuronal milieu.

## Conclusion

The present study highlights the impact of arginase 2 deficiency in reducing cellular infiltrates, attenuating the activation of microglia and macrophages, mitigating axonal and myelin damage, and reducing astrogliosis in an experimental model of optic neuritis. Therefore, arginase 2 deficiency or blockade may positively contribute towards targeted neuroprotection in MS-induced optic neuritis.
